# Rapid Solidification Brings Ultralow Thermal Conductivity and Superior Thermoelectric Performance for Intrinsic GeTe Alloy

**DOI:** 10.1002/advs.78267

**Published:** 2026-10-27

**Authors:** Guanghui Liu, Shuangxiong Ma, Xintong Zhang, Dou Li, Weili Wang

**Affiliations:** ^1^ School of Physical Science and Technology Northwestern Polytechnical University Xi'an People's Republic of China

**Keywords:** intrinsic GeTe, liquid undercooling, rhombohedral‐cubic, thermoelectricity

## Abstract

Intrinsic GeTe suffers from excessively high carrier concentrations caused by Ge vacancies and limitations in phase regulation, which constrain further enhancement of its thermoelectric performance. In this work, rapid solidification with glass fluxing techniques is employed to achieve a large undercooling of Δ*T* = 112–122 K in the GeTe alloy for the first time. By Ge enrichment, the carrier concentration is tuned to an optimal range of 5.7 × 10^20^ cm^−3^ and the carrier mobility is enhanced to 75.3 cm^2^ V^−1^ s^−1^. The stoichiometric GeTe alloy retains a portion of the high‐temperature cubic phase at room temperature, forming a rhombohedral‐cubic (α+β) uniform dual‐phase structure with a specific crystallographic orientation. DFT calculations of the band structure, density of states, and phonon spectrum reveal the influence of the α+β structure on electrical transport and thermal diffusion. This unique microstructure combined with multiscale defect engineering enables a high figure of merit (*zT*) of 1.32 at 700 K for the Ge_1.03_Te alloy, with a total processing time of less than 2 h, significantly improving the preparation efficiency. This work demonstrates that liquid undercooling technology is an efficient preparation method for effectively regulating the phase composition and microstructure of thermoelectric alloys.

## Graphical abstract

Rapid solidification with glass fluxing is first applied to the intrinsic GeTe alloy and achieves large undercooling within 2 h. This enables partial retention of the high‐temperature cubic phase at room temperature and forms a crystallographically oriented α+β dual‐phase structure and a hierarchical defect network. These features synergistically weaken electron‐phonon coupling and yield a peak *zT* of 1.32 at 700 K.

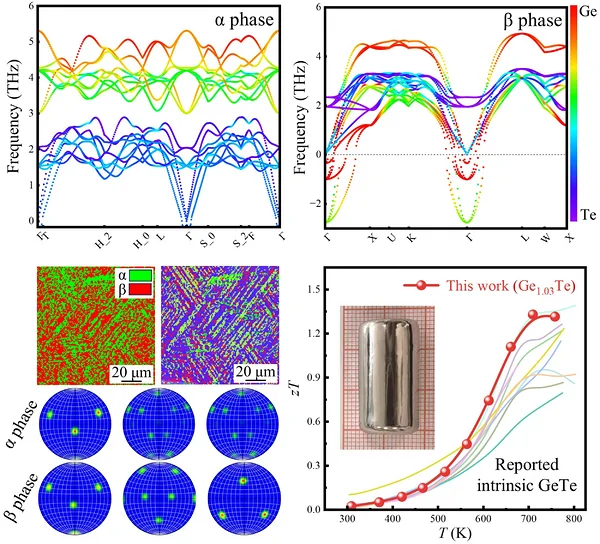

## 1 Introduction

Thermoelectric materials enable direct mutual conversion between thermal and electrical energy, providing an important technological pathway for addressing global energy and environmental challenges in waste heat recovery and solid‐state cooling [1, 2]. Their operating principle is based on the Seebeck effect, the Peltier effect, and the Thomson effect, which together form the foundation for thermoelectric power generation and multistage cooling [3, 4, 5]. Currently, persistently superior thermoelectric materials figures of merit (*zT*) have been reported in mid‐to‐low temperature thermoelectrics such as GeTe, PbTe, SnTe, skutterudites, and chalcogenides [6, 7, 8, 9, 10]. The GeTe alloy is a self‐doped p‐type narrow‐bandgap semiconductor [11, 12], and its thermoelectric performance is mainly determined by the dimensionless *zT* =  (*S* ^2^σ*T*)/(κ_*e*_ + κ_*l*_), where *S* is the Seebeck coefficient, *σ* is the electrical conductivity, *T* is the absolute temperature, and κ_*e*_ and κ_*l*_ are the electronic and lattice thermal conductivities, respectively [13, 14]. The advantage of the intrinsic GeTe alloy originates from its large Grüneisen parameter and anharmonic lattice dynamics, which provide the potential to achieve intrinsically low lattice thermal conductivity [15, 16]. However, it also faces several intrinsic challenges. For one thing, multiple intrinsic point defects exist in the GeTe alloy, including Ge vacancies (V_Ge_), Te vacancies (V_Te_), Ge antisite (Ge_Te_), and Te antisite (Te_Ge_) [17]. Among them, V_Ge_ has the lowest formation energy and acts as the dominant defect in the intrinsic GeTe alloy. This leads to a hole concentration as high as approximately 10^21^ cm^−3^, which far exceeds the optimal carrier concentration range [18]. For another, the α phase exhibits a larger energy offset between the primary valence band L band and the secondary valence band Σ band than the β phase does. This substantial offset impedes the effective participation of the secondary Σ band in electrical transport, thereby restricting the Seebeck coefficient and power factor [19, 20].

In the undoped or lightly doped GeTe alloy, the Seebeck coefficient is strongly dependent on the carrier concentration, and an excessively high hole concentration significantly reduces it [21]. Meanwhile, the volatilization of Te during preparation causes deviations from the initial stoichiometric ratio, making precise control of the carrier concentration difficult. Mainstream strategies largely rely on doping engineering, such as donor doping (Bi, Sb, etc.) or alloying regulation (CuSbSe_2_, AgSbTe_2_, etc.), to suppress the formation of excess V_Ge_ [22, 23, 24, 25]. The core mechanism of these strategies is that additional dopant atoms fill V_Ge_ or alter the defect equilibrium, thereby reducing the sources of vacancy generation to a certain extent. Despite the significant progress achieved by these strategies, precise regulation of carrier concentration solely through intrinsic defect control and preparation process optimization without introducing foreign dopant elements still faces considerable limitations [26]. Additionally, the GeTe alloy possesses a highly symmetric face‐centred cubic rock‐salt (β phase) structure at elevated temperatures and undergoes a displacive solid‐state phase transition at about 700 K, transforming into a rhombohedral (α phase) structure upon slow cooling to room temperature [27, 28]. This phase transition involves no long‐range atomic diffusion and only a cooperative atomic displacement along the <111> direction, and it is therefore intrinsically reversible [29, 30, 31]. Owing to its higher crystal symmetry, the β phase exhibits a smaller energy offset between the L point and the Σ point in the valence band, which enhances band degeneracy and leads to superior electrical transport performance [19, 32]. However, the intrinsic GeTe alloy obtained by conventional preparation methods typically exist as a single α phase at room temperature, and doping, alloying, or entropy engineering is generally required to retain a portion of the β phase.

In solid materials, the total thermal conductivity (κ_*tot*_) generally consists of three contributions, namely electronic (κ_*e*_), lattice (κ_*l*_), and bipolar thermal conductivity (κ_*b*_) [33]. For degenerate p‐type semiconductors with high carrier concentrations such as the GeTe alloy, the contribution of bipolar thermal conductivity is relatively limited, and the κ_*tot*_ is still mainly determined by κ_*e*_ and κ_*l*_ [21]. The Wiedemann‐Franz law states that κ_*e*_  =  *L*σ*T*, where *L* is the Lorenz number, indicating a strong coupling between κ_*l*_ and electrical conductivity (*σ*) [34, 35]. The ideal goal for thermoelectric performance optimization is to substantially reduce the κ_*l*_ without significantly sacrificing the *σ* [25]. Notably, the charge carrier concentration has a direct and simultaneous influence on both parameters. This inherent contradiction indicates that carrier concentration engineering alone is difficult to simultaneously satisfy the requirements of high electrical conductivity and low thermal conductivity [36]. It is urgent to explore microstructural design strategies that can independently regulate electron and phonon transport, simultaneously enhance the power factor and reduce the lattice thermal conductivity, thereby improving the *zT* value. Current research focuses mainly on enhancing thermoelectric performance through doping, alloying, and entropy engineering, while preparation methods remain largely limited to zone melting and ball milling combined with sintering [23, 24, 37]. It has been reported that non‐equilibrium rapid synthesis methods such as self‐propagating high‐temperature synthesis (SHS) can enable the coexistence of α+β phases in the tine GeTe alloy at room temperature, thereby suppressing electron‐phonon coupling [38]. However, subsequent ball milling and sintering steps are usually required, and impurities are inevitably introduced during prolonged ball milling. Therefore, there is an urgent need to explore a novel, simple, and efficient preparation method that can simultaneously achieve phase composition regulation, multiscale defect construction, and weakened electron‐phonon transport coupling while avoiding impurity introduction and air oxidation.

This work first employs rapid solidification combined with glass fluxing to prepare the GeTe alloy. The core physical principle lies in the use of molten B_2_O_3_ glass encapsulation to suppress or eliminate heterogeneous nucleation sites within the melt, significantly delaying the nucleation process and achieving a large undercooling of Δ*T* = 112–122 K far below the equilibrium solidification temperature. This non‑equilibrium solidification process brings about some distinctive effects. On one hand, the large undercooling suppresses the complete β to α phase transformation, allowing a portion of the high‐temperature face‐centered cubic (β) phase to be retained at room temperature and to coexist with the low‐temperature rhombohedral (α) phase, forming an α+β dual‐phase structure with a specific crystallographic orientation relationship. First‐principles calculations indicate that the introduction of the β phase brings two independent electronic structure effects, which involve increasing valley degeneracy and modifying band‐edge dispersion to reduce the density‐of‐states effective mass, thereby synergistically optimizing electrical transport properties. On the other hand, the large undercooling generates abundant dual‐phase interfaces, lattice distortions, and microscopic defects, constructing a hierarchical phonon scattering network across multiple length scales. This network broadly suppresses the entire phonon spectrum, thereby significantly reducing the lattice thermal conductivity.

## 2 Results and Discussion

Figure S1 illustrates the preparation of the intrinsic GeTe alloy for the first time by rapid solidification combined with glass fluxing technologies [39, 40]. Literature reports identify tuning the Ge/Te ratio as one of the effective strategies to optimize carrier concentration and enhance the power factor [41, 42]. Therefore, three GeTe alloy compositions, GeTe‐1, GeTe‐2, and GeTe‐3, are selected from the binary phase diagram in Figure 1a and Table S1 [43]. For comparison, a stoichiometric GeTe alloy, denoted as GeTe‐0, is prepared under near‐equilibrium solidification conditions. The equilibrium phase diagram indicates that the GeTe‐0 alloy first forms a cubic (β) phase when the temperature decreases to 998 K. Upon further cooling to approximately 700 K, the Peierls mechanism triggers a displacive solid‐state phase transition. This transition originates from the susceptibility of the half‐filled p‐orbital band to Peierls‐type instability, which leads to a cooperative displacement of atoms along the <111> direction, thereby forming alternating long and short Ge─Te bonds [31]. A slight tensile lattice distortion accompanies this process, and the phase eventually transforms into a rhombohedral (α) phase [30]. The undercooling is defined as Δ*T* = *T_L_*  − *T_N_*, where *T_L_* and *T_N_* are the equilibrium liquidus temperature of the material and the temperature at which the melt actually begins to nucleate, respectively. The preparation process is monitored and recorded in real time using a Sensor Therm‐GmbH M316 monochromatic infrared pyrometer with non‐contact measurement capability. Under rapid solidification, the encapsulation by molten B_2_O_3_ glass suppresses or eliminates heterogeneous nucleation sites within the melt and significantly delays the nucleation process. This enables solidification far below the liquidus temperature, thereby achieving large undercooling, which reaches as high as 112 K for the GeTe‐1 alloy (Figure 1b). The strong recalescence induced by the large undercooling suppresses the complete β to α phase transformation, so that a portion of the β phase is retained at room temperature, subsequently forming an (α+β) dual‐phase structure. Unlike thermodynamic methods that rely on doping or alloying to regulate phase transitions, this study adopts a kinetically driven purely physical strategy.

**FIGURE 1:**
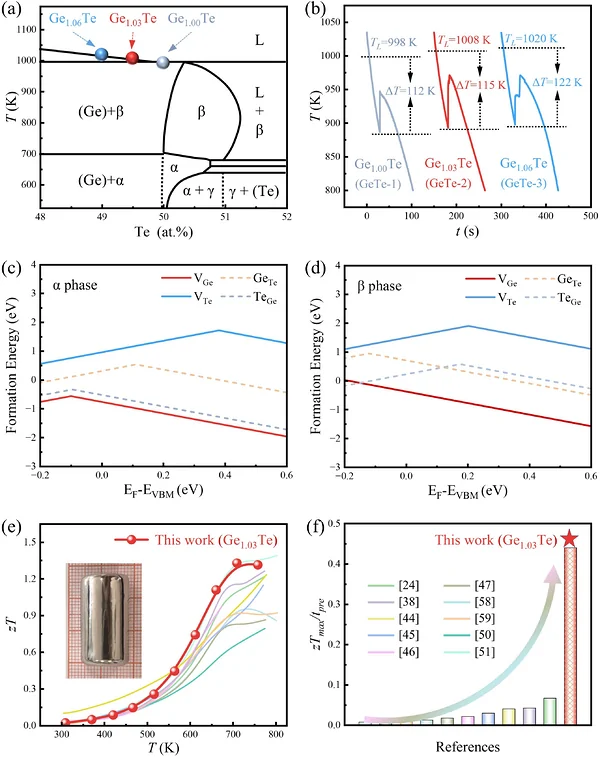
Preparation and thermoelectric properties. (a) Equilibrium solidification phase diagram. (b) Rapid solidification curves. (c,d) The formation energy of atomic defects in α and β phases as a function of the relative Fermi energy under the Ge‐rich condition; here VBM refers to the valence band maximum. (e) Figure of merit (*zT*) values (Inset graph of the actual alloy) and (f) the efficiency plot of the *zT* to preparation‑time ratio of the GeTe‑2 alloy.

Ge‐rich environment increases the Ge vacancies (V_Ge_) formation energy and suppress V_Ge_ formation, thereby weakening the stabilization effect of V_Ge_ on the β phase. DFT calculations also confirm a lower V_Ge_ formation energy in the β phase than in the α phase in Figure 1c,d, providing direct thermodynamic evidence for this effect, consistent with literature reports [17]. Since their compositions are close to the stoichiometric ratio of the GeTe‐2 and GeTe‐3 alloy, the primary phase is the β matrix phase that precipitates first. After reaching the eutectic temperature, a small amount of (Ge)+β eutectic forms, which is consistent with the bimodal feature of the solidification curve. Upon further cooling, a solid‐state phase transition occurs, so that part of the β phase is retained to room temperature, ultimately forming a small volume fraction of (Ge)+(α+β) eutectic distributed at the grain boundaries of the α+β matrix phase. Fine tuning of the Ge content synergistically optimizes the power factor (*PF*) and total thermal conductivity (κ_*tot*_) while weakening the coupling between electron and phonon transport, thus enabling the intrinsic Ge_1.03_Te alloy to achieve an excellent figure of merit (*zT*) value of 1.32 (Figure 1e). This value is comparable to the highest values obtained by state‐of‐the‐art processing routes such as ball milling refinement, spark plasma sintering, and prolonged annealing [24, 38, 44, 45, 46, 47, 48, 49, 50, 51]. Moreover, the increased pressure generated by the molten B_2_O_3_ glass on top of the sample significantly enhances the alloy density. Figure 1f shows that the entire preparation process can be completed within 2 h, representing a significant improvement in preparation efficiency compared with the current mainstream methods. These results indicate that this method holds potential for preparing thermoelectric materials, providing an innovative technique for the subsequent development of efficient processing routes and large‐scale industrial production.

Electron backscatter diffraction (EBSD) is further employed to analyze the phase composition of the GeTe alloy, thereby clarifying the influence mechanism of microstructure on electron and phonon transport properties. The phase map and inverse pole figure (IPF) in Figure 2a,b jointly reveal that the GeTe‐1 alloy with a large undercooling of Δ*T* = 112 K exhibits an α+β dual‐phase structure, in which the two phases are interwoven and uniformly distributed. The key physical mechanism lies in the rapid solidification process. When the undercooled melt nucleates, the system directly precipitates the high‐temperature β phase from the liquid. Subsequently, the violent release of latent heat of crystallization triggers strong recalescence. Because the recalescence process is extremely short and the subsequent cooling rate is extremely fast, long‐range atomic diffusion is severely inhibited. The cooperative atomic displacement required for the β→α displacive phase transition cannot be fully completed. As a result, part of the β phase is frozen to room temperature and coexists with the α phase to form an α+β dual‐phase structure. Both phases exhibit a specific crystallographic orientation relationship (Figure 2c), namely <111>β∥<0001>α. During the phase transition, atoms in the β phase undergo a cooperative displacement along the <111> direction of the cubic unit cell, which transforms into the polar axis <0001> direction of the α phase, so that the two phases naturally inherit the above orientation relationship [52]. Meanwhile, the viscous B_2_O_3_ glass forms a negative pressure cavity above the melt, which effectively suppresses splashing and boiling of the melt. A relatively stable solidification environment is established, ensuring that the preferential orientation growth process is not disturbed. Repeated experiments demonstrate that this phenomenon is not accidental but results from the synergistic effect of interfacial strain energy minimization, the kinetic pathway of the displacive phase transition, and the intrinsic crystallographic characteristics of the material. As the Ge content increases, the α phase fraction rises from 42.2% to 58.2%, indicating that excess Ge destabilizes the β phase at room temperature (Figure S2).

**FIGURE 2:**
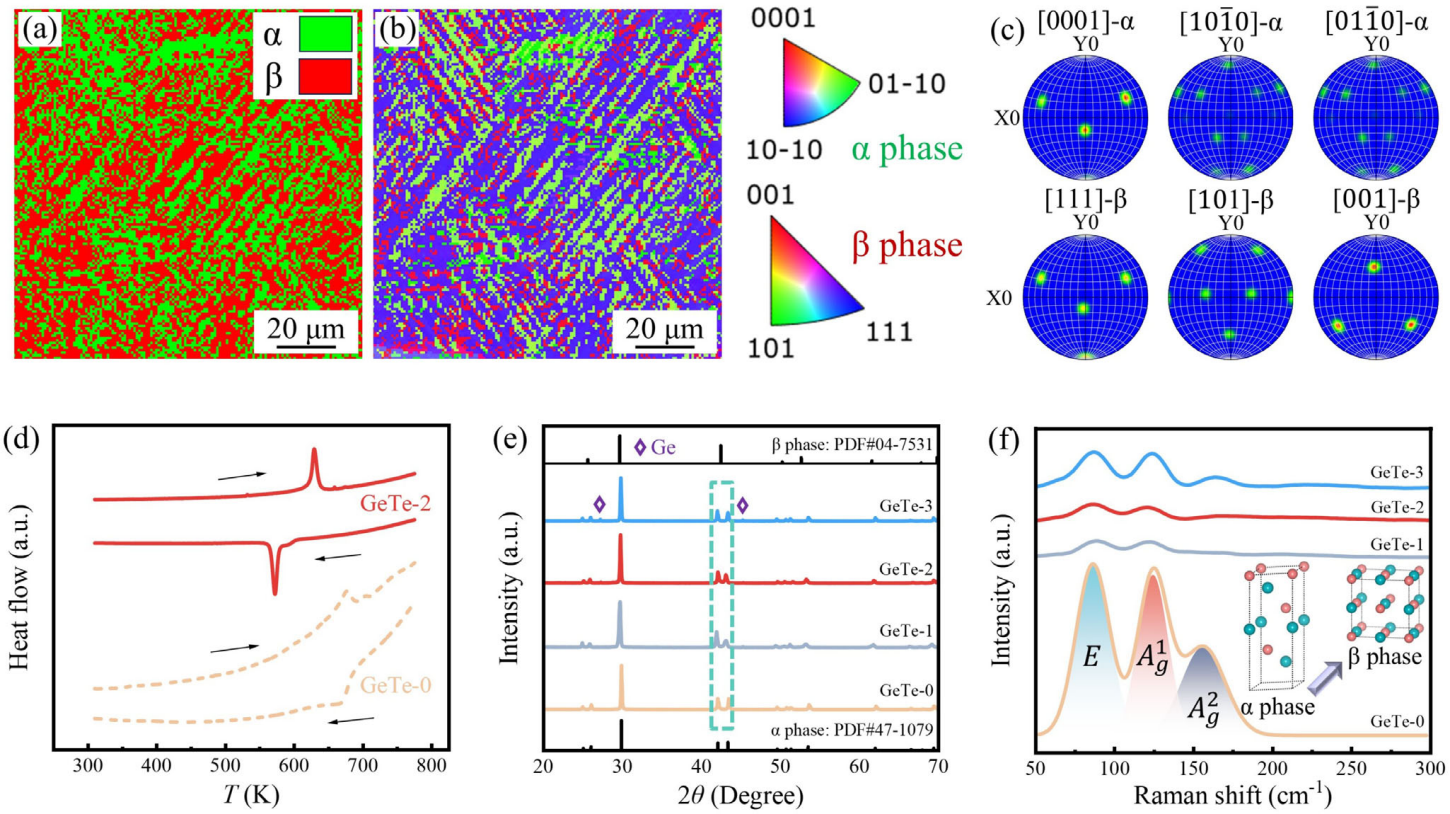
Phase composition identification. EBSD micrographs of (a) phase map, (b) IPF map, and (c) pole maps in the GeTe‐2 alloy. (d) DSC curves, (e) XRD, and (f) Raman spectra in the GeTe alloy powder.

To reveal the influence of strong recalescence induced by large undercooling on suppressing the complete transformation of the β phase, differential scanning calorimetry (DSC) measurements are conducted, with the results depicted in Figure 2d. Compared with the near‐equilibrium solidified GeTe‐0 alloy, the solid‐state phase transition temperatures of the rapidly solidified GeTe‐2 alloy shift to lower temperatures during both heating and cooling. Moreover, after 5 thermal cycles over a wide temperature range, the phase transition temperatures remain unchanged (Figure S3). This observation indicates that if the retention of the β phase were solely caused by a fast cooling rate, the retained β phase should undergo the α→β transition at a temperature close to the equilibrium phase transition temperature of approximately 630 K during subsequent DSC heating. However, the experimentally observed phase transition temperature is approximately 570 K, much lower than the equilibrium value. This result arises because the latent heat released by strong recalescence generates intense thermal stress and lattice distortion in the newly formed β phase prior to the solid‐state phase transition. Given that the lattice distortion between the α and β phases of the GeTe alloy is only about 1%–2%, such pre‐existing distortion may make it difficult for the subsequent partial β phase to further transform into the α phase. Meanwhile, EBSD characterization is performed on the cycled sample. The results show no significant changes in either the phase composition or the microstructure (Figure S4). This indicates that the dual‐phase microstructure exhibits good thermal stability during repeated heating and cooling. To further elucidate the composition‐induced phase evolution, room‐temperature X‐ray diffraction (XRD) analysis is performed on the powdered alloys (Figure 2e). All diffraction peaks of the GeTe‐0 alloy are well indexed to the α phase structure (PDF#47‐1079), and symmetric double peaks are observed in the magnified characteristic peaks within the 41°–44° range (Figure S5). For the rapidly solidified GeTe‐1 alloy, when the α and β phases are highly interwoven at the micro/nanoscale with a specific crystallographic orientation relationship, the XRD peaks do not split into three separate peaks but instead appear as broadened and asymmetric double peaks, consistent with literature reports [53]. As the Ge content increases, the asymmetry weakens, and the Ge characteristic peaks at ∼27° and ∼45° intensify, indicating that excess Ge is unfavorable for suppressing the β to α transformation.

Raman mapping may offer a viable route for spatially resolving the α and β phase composition, as depicted in Figure 2f. The typical Raman modes of room‐temperature α‐GeTe are the E mode (about 80–90 cm^−1^) and the A_g_ mode (approximately 120–125 cm^−1^) [54]. As the temperature increases, the frequencies of both modes decrease (red‐shift), primarily arising from anharmonic phonon‐phonon interactions and accompanied by thermal expansion effects that manifest as negative thermal expansion near the phase transition [55]. Since the β phase is Raman‐inactive, the composition analysis of the α+β dual‐phase mainly relies on monitoring the intensity attenuation of the α phase characteristic peaks. Compared with the GeTe‐0 alloy, the rapidly solidified GeTe‐1 alloy exhibits a significantly reduced peak intensity, indicating that part of the β phase is retained at room temperature. However, with increasing Ge content, the peak intensity gradually increases again, suggesting that excess Ge is unfavorable for the retention of the β phase at room temperature. In addition, the GeTe alloy is extremely sensitive to oxygen, and its surface oxidation characteristics are one of the key factors determining device performance and stability. To evaluate whether oxidation occurs in the prepared GeTe alloy during preparation, X‐ray photoelectron spectroscopy (XPS) is employed to analyze the surface chemical composition and bonding states of the alloy powder (Figure S6a). Survey scans detect Ge 2p, Ge 3d, Te 3d, C 1s, and O 1s signals within the range of 50–1300 eV, consistent with typical GeTe characteristic peaks. In the high‐resolution Ge 2p spectrum, four characteristic peaks are observed. The peaks located at 1218.0 and 1249.0 eV are assigned to the 2p_3_/_2_ and 2p_1_/_2_ of Ge^2^^+^ in the GeTe alloy. As illustrated in Figure S6b, the peaks at 1221.2 and 1252.2 eV are attributed to the 2p_3_/_2_ and 2p_1_/_2_ of Ge^4^^+^ in GeO_2_ [56]. However, only two peaks at 572.8 and 583.3 eV are detected in the high‐resolution Te 3d spectrum, corresponding to the 3d_5_/_2_ and 3d_3_/_2_ of Te^2−^ in the GeTe alloy (Figure S6c). No characteristic peak of TeO_2_ (Te^4+^) is observed near 576 eV. These results indicate that although preferential oxidation of Ge occurs on the GeTe alloy surface with the formation of GeO_2_, the chemical state of Te remains stable and is not oxidized [57]. Therefore, a slight degree of surface Ge oxidation exists in the alloy during preparation and exposure.

Scanning electron microscopy (SEM) is employed to investigate the microstructural evolution of the GeTe alloy with different compositions. Figure S7 shows that all rapidly solidified alloys with large undercooling exhibit a dual‐phase α+β structure, and the volume fraction of (Ge)+(α+β) eutectic gradually increases with increasing excess Ge content. Taking the GeTe‐2 alloy with Δ*T* = 115 K as an example, SEM images and corresponding energy‐dispersive X‐ray spectroscopy (EDS) maps in Figure 3a,b reveal a dominant α+β matrix phase together with a small amount of uniformly distributed network‐like (Ge)+(α+β) eutectic. An appropriate amount of eutectic assists in scattering low‐frequency phonons, whereas excessive eutectic forms a coarsened high thermal conductivity network that provides fast channels for phonon transport and conversely increases the lattice thermal conductivity (κ_*l*_). Therefore, the microstructure of the GeTe‐2 alloy weakens electron‐phonon coupling and achieves a balance between a high *PF* and a low κ_*l*_. Aberration‐corrected transmission electron microscopy (TEM) is employed to further elucidate the microstructural origin of the reduced thermal conductivity and to confirm the coexistence of dual phases at room temperature. Samples are prepared by focused ion beam (FIB) milling (Figure S8). As shown in Figure 3c, the high‐resolution STEM image displays a typical “herringbone” microstructure [58, 59]. Such domain structures are common in ferroelectric crystals, where the black‐and‐white contrast arises from differences in crystallographic axis orientation. Under two‐beam conditions, the contrast across the domain interfaces exhibits mirror symmetry, a characteristic feature of twin structures originating from the displacive phase transition from β to α during solidification. In addition, EDS analysis confirms uniform distribution of Ge and Te throughout the GeTe‐2 alloy (Figure S9). The TEM image in Figure 3d shows a lattice spacing of 0.21 nm, corresponding to the (220) plane. As presented in the magnified region of Figure 3e, the lattice fringes exhibit irregular distortion, which results from dislocations and lattice distortions generated during non‐equilibrium solidification. Strain distribution maps along the *xy* direction obtained by inverse fast Fourier transform (IFFT) and geometric phase analysis (GPA) reveal dense dislocations and pronounced strain fluctuations caused by dislocations and planar defects. The selected area electron diffraction (SAED) pattern in Figure 3f is indexed along the [$\bar{1}$11] zone axis. These microstructural features serve as effective scattering sources for mid‐ and high‐frequency phonons, significantly lowering the lattice thermal conductivity (κ_*l*_).

**FIGURE 3:**
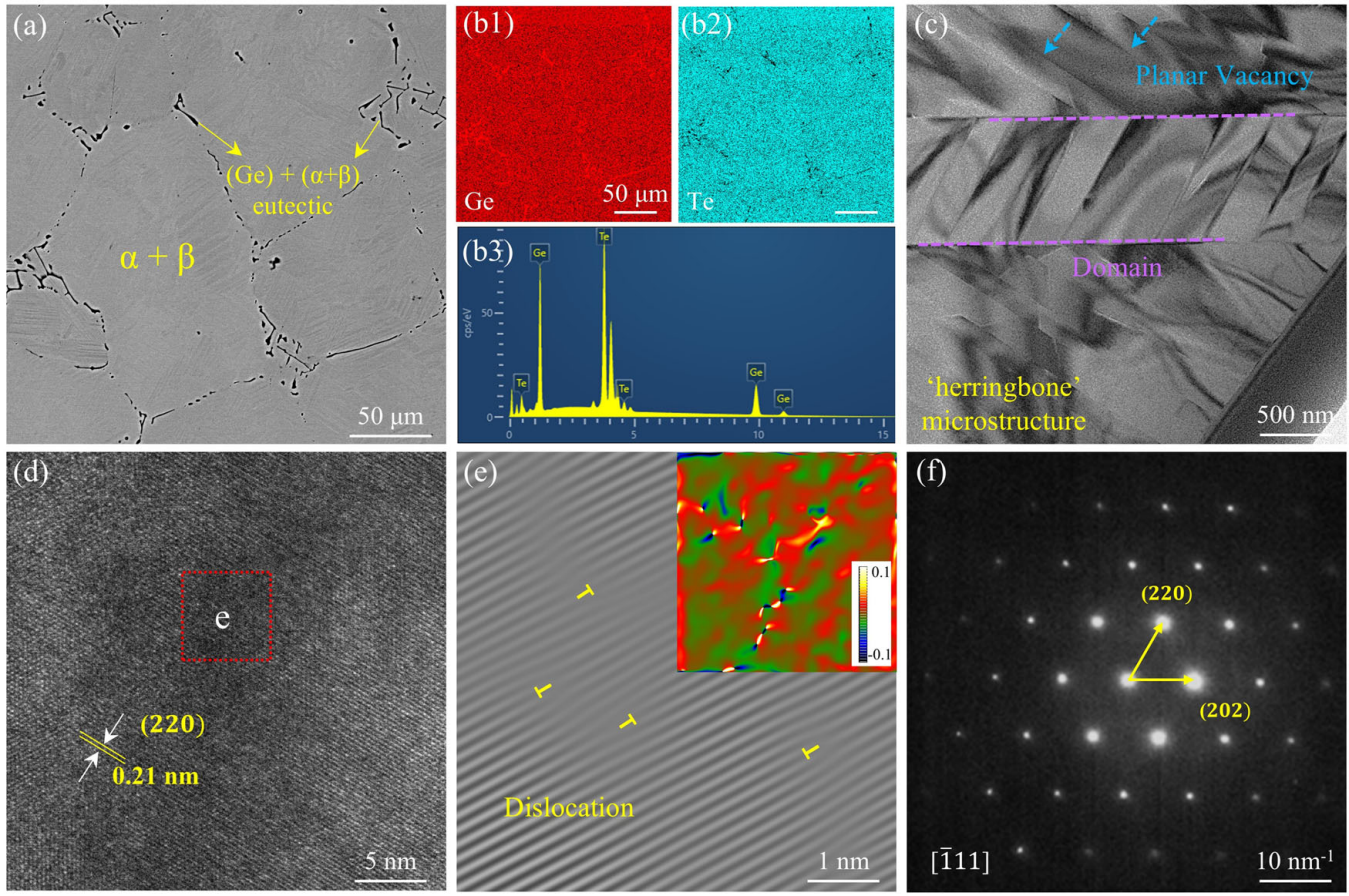
Microstructural characterization. (a) SEM image, (b) EDS spectrum, (c) TEM image, (d) HAADF image, (e) IFFT square region in d. pattern, with an inset showing the *xy* direction confirmed by GPA, and (f) Indexed SAED pattern of β phase of the GeTe‐2 alloy.

To elucidate the influence of the α+β dual‐phase structure induced by large undercooling on electrical transport properties, density functional theory (DFT) calculations are performed to obtain the corresponding electronic band structures and density of states (DOS). Figure 4a,b and Figure S10 show that the β phase possesses a narrower theoretical band gap and higher symmetry than the α phase, thereby generating more equivalent points in reciprocal space. This enhanced symmetry increases the valley degeneracy (*N_v_*) relative to the α phase. It should be noted that an increase in valley degeneracy does not directly reduce the band‐edge effective mass of a single band. Valley degeneracy and effective mass are two independent concepts. The former depends on the number of equivalent valleys in reciprocal space, while the latter depends on the curvature of the band edge. Pisarenko analysis indicates that the apparent density‐of‐states effective mass of the rapidly solidified sample decreases from 1.48 *m*_0_ to 1.12 *m*_0_ (Figure S11). This change mainly arises from a substantial reduction in carrier concentration. The decrease in carrier concentration shifts the Fermi level deeper into the valence band, altering the relative contributions of the L band and Σ band to electrical transport and weakening the non‐parabolic band effect, thereby reducing the effective mass obtained from the Pisarenko fitting [60, 61]. Therefore, this decrease in effective mass is not directly caused by the multi‐valley band structure of the β phase or by the flatness of the density of states itself. The α+β dual‐phase structure enhances valley degeneracy and reduces the density‐of‐states effective mass through parallel transport channels and the high symmetry of the β phase, thereby improving carrier mobility while maintaining the Seebeck coefficient and weakening the coupling between electrical conductivity (*σ*) and the Seebeck coefficient (*S*).

**FIGURE 4:**
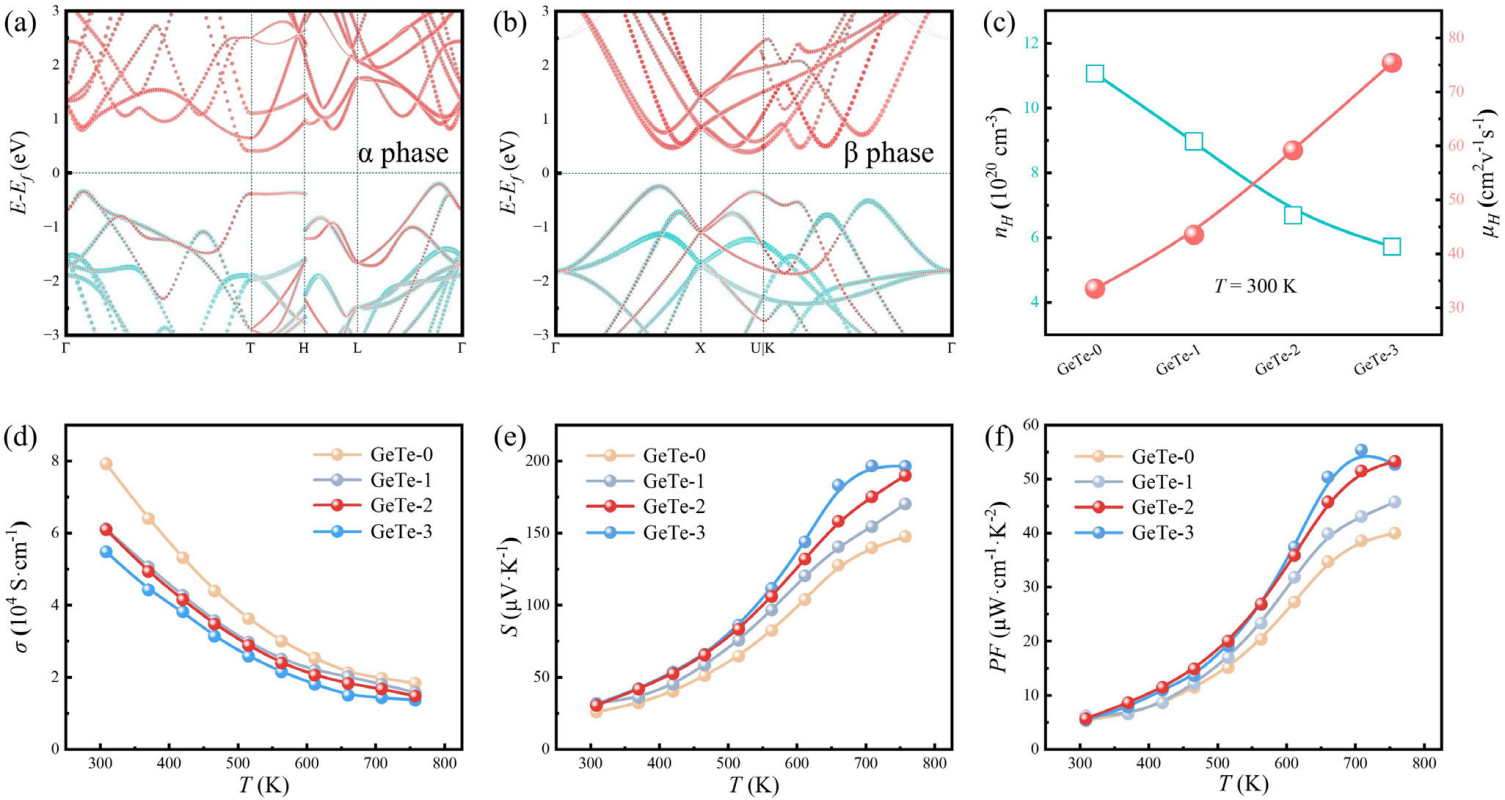
Electrical transport properties. Energy band of the (a) α phase and (b) β phase. (c) Hall effect at room temperature, (d) Electrical conductivity (*σ*), (e) Seebeck coefficient (*S*), and (f) Power factor (*PF*) of the GeTe alloy.

As illustrated in Figure S12, differential charge density calculations reveal that localized charge redistribution at the dual‐phase interface modifies interatomic force constants and enhances mid‐frequency phonon scattering. However, because no long‐range electrostatic field is formed, the scattering effect on charge carriers remains limited, which facilitates weakened coupling where phonons are effectively scattered while electron transport is not significantly affected. In Figure 4c, room‐temperature Hall measurements demonstrate that as the Ge/Te ratio increases from 1:1 to 1.06:1, the carrier concentration (*n*) decreases by nearly one order of magnitude from 1.11 × 10^21^ to 5.72 × 10^20^ cm^−3^, whereas the mobility µ increases to 75.34 cm^2^ v^−1^ s^−1^ This improvement originates from the suppression of V_Ge_ formation and the weakened point‐defect scattering. The resistivity exhibits metallic behavior and decreases with increasing temperature. Room‐temperature electrical conductivity (*σ*) decreases from 7.92 × 10^4^ to 5.48 × 10^4^ S·cm^−1^ in Figure 4d, mainly due to the reduction in *n*, while the dual‐phase structure with high‐density interfaces stabilizes *σ* at elevated temperatures. The Seebeck coefficient (*S*) is enhanced by the combined effects of V_Ge_ filling and the dual‐phase structure, rising from 147.59 to 189.89 µV·K^−1^ at 750 K (Figure 4e). Consequently, the power factor (*PF*) of the GeTe‐2 alloy reaches a maximum of 53.24 µW·cm^−1^·K^−2^ at 750 K (Figure 4f).

In typical GeTe‐based IV‐VI thermoelectric materials, the α and β phases exhibit distinctly different phonon spectral characteristics that profoundly influence thermal conductivity. Therefore, phonon dispersion calculations are performed using the density functional perturbation theory (DFPT) method [62]. Figure 5a–c shows that the phonon dispersion relations of the α phase remain real‐valued across the entire Brillouin zone, indicating intrinsic dynamical stability. At the Γ point, six vibrational modes exist, and a clear frequency gap separates the three acoustic branches from the three optical branches. The stereochemical activity of the Ge 4s^2^ lone pair electrons induces a Peierls distortion along the <111> direction, leading to periodic variation in Ge‐Te bond lengths. This symmetry breaking introduces fluctuations in bond lengths and force constants. In contrast, the β phase is intrinsically dynamically unstable at low temperatures, with imaginary‐frequency soft modes appearing near the Γ point. It should be noted that the appearance of imaginary frequencies in the DFPT calculations for the β phase is not a computational error or methodological flaw but a direct reflection of the intrinsic lattice instability of this material, consistent with literature reports [19, 48]. Apart from the imaginary‐frequency region, the phonon group velocity of the β phase is generally lower than that of the α phase, which is attributed to its higher coordination number and longer Ge─Te bonds (Figure 5d). Meanwhile, its anharmonic scattering rate is significantly higher than that of the α phase. Overall, the static structural modulation and bond length fluctuations caused by Peierls distortion in the α phase and the strong anharmonic scattering induced by dynamic soft modes in the β phase cover different frequency ranges. When the two phases coexist at the micro/nanoscale, the phase boundaries become additional strong scattering centers.

**FIGURE 5:**
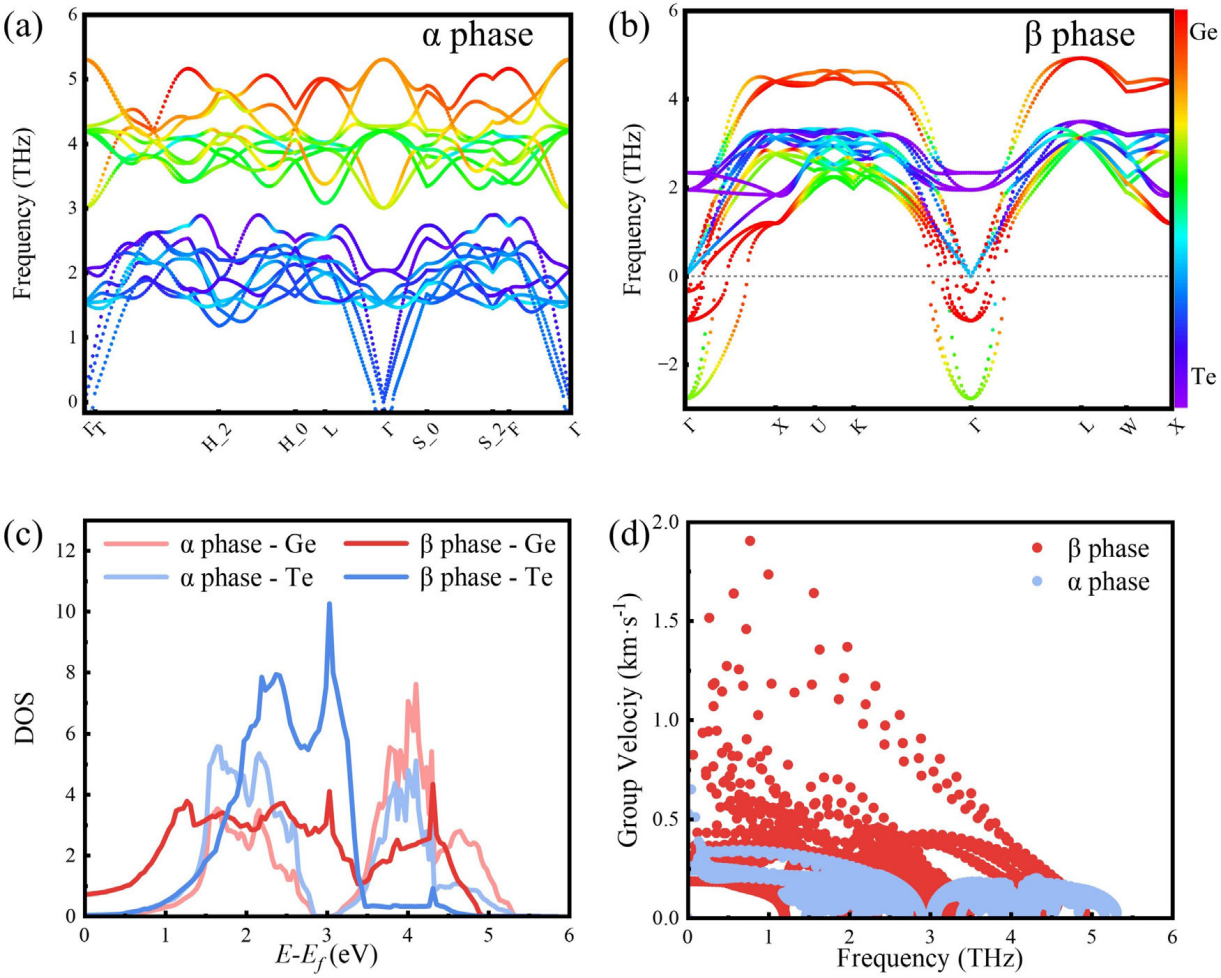
Phonon dispersion calculations based on density functional perturbation theory (DFPT). (a,b) Phonon dispersion spectra, (c) density of states (DOS), and (d) Frequency dependence of the group velocities for the α and β phases.

Figure 6a shows that the GeTe‐2 alloy exhibits the lowest total thermal conductivity of approximately 2.69 W·m^−1^·K^−1^ at 700 K. Over the entire temperature range, the total thermal conductivity first decreases and then increases, which is attributed to the temperature‐induced strengthening of Ge─Ge and Te─Te second‐nearest‐neighbor bonds near 700 K [63]. According to the Wiedemann–Franz law, the electronic thermal conductivity κ_*e*_ is calculated by κ_*e*_  =  *L*σ*T*, where the Lorenz number (*L*) is estimated from the experimentally measured Seebeck coefficient using the widely adopted empirical formula [17, 61, 64]. This method is based on the single parabolic band model and the acoustic phonon scattering assumption and has been widely applied in GeTe‐based thermoelectric systems. The present work uses the measured *S*(*T*) at each temperature point to calculate the corresponding temperature‐dependent *L* rather than assuming a constant metallic limit (Table S2). The electronic thermal conductivity of the GeTe‐2 alloy decreases from 5.98 to 4.40 W·m^−^^1^·K^−^^1^ at 300 K, as displayed in Figure S13. This reduction mainly results from the filling of *V_Ge_* in the Ge‐rich environment, which significantly lowers the hole concentration. Moreover, its lattice thermal conductivity (κ_*l*_) reaches as low as 0.62 W m^−^^1^ K^−^^1^ at 700 K (Figure 6b). Figure 6c and Table S3 present the Debye‐Callaway model fitting results for the κ_*l*_ at 300 K, considering grain boundary scattering (B), Umklapp scattering (U), dislocation scattering (D), and point defect scattering (PD) [18, 24]. It should be noted that this model aims to semi‐quantitatively evaluate the relative contributions of each scattering mechanism rather than provide precise quantitative predictions. The introduction of each scattering term in the model is not arbitrary or purely mathematical fitting but is based on published references and analysis of microstructural characterization results, ensuring physical reliability.

**FIGURE 6:**
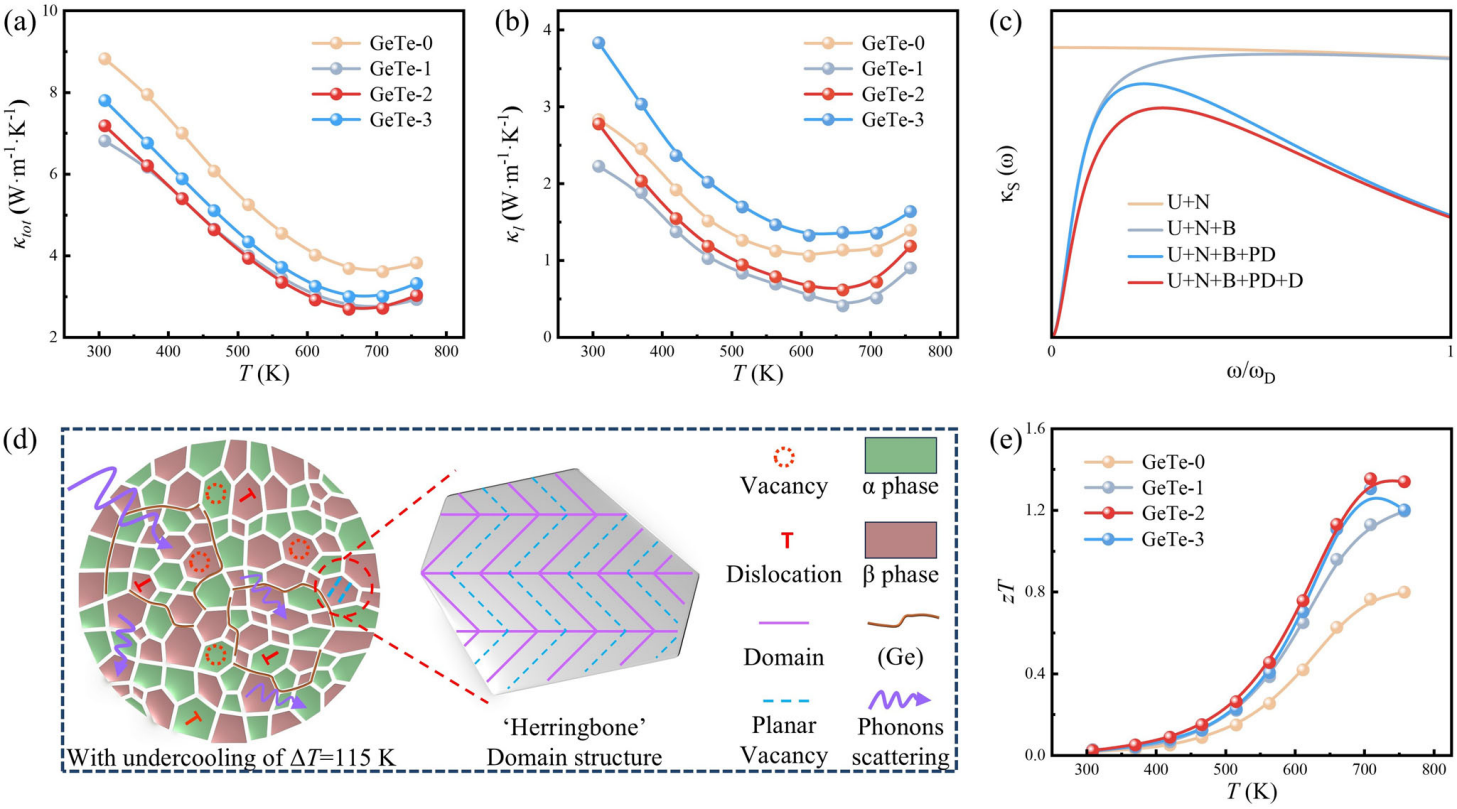
Thermal transport properties. (a) Thermal conductivity (κ_*tot*_), (b) Lattice thermal conductivity (κ_*l*_), (c) Calculated κ_*s*_ using the Debye–Callaway model at 300 K, (d) Schematic diagram of solidification morphology and its phonon scattering, and (e) Figure of merit (*zT*) of the GeTe alloy.

The analysis indicates that the multiscale defects introduced by large undercooling during rapid solidification, combined with glass fluxing, act synergistically on phonon scattering. Point defect scattering mainly affects high‐frequency phonons, dislocation and twin boundary scattering contribute significantly to mid‐frequency phonons, and grain boundary and phase boundary scattering effectively suppress low‐frequency phonons. These scattering mechanisms collectively construct a phonon scattering network covering the full frequency range. This network is consistent with the α+β dual phase confirmed by EBSD micrographs and the dense dislocations, twins, and lattice distortions observed by TEM images at the dual‐phase interfaces. Nevertheless, excess Ge suppresses the formation of V_Ge_, thereby weakening the scattering of high‐frequency phonons by point defects, while the numerous coarsened eutectic networks formed by Ge segregation provide high thermal‐conductivity fast channels for phonon transport. Together, these two effects cause the κ_*l*_ of the GeTe‐3 alloy to increase rather than decrease. Benefiting from the comprehensive balance between electrical transport and thermal diffusion in the Ge‐rich environment, the GeTe‐2 alloy achieves a peak *zT_max_* of 1.32 at 700 K and an average *zT_ave_* of 0.56 over the temperature range of 300–750 K (Figure 6e and Figure S14). Furthermore, the correlation among Δ*T*, β phase fraction, microstructure, and thermoelectric performance is further established using the GeTe‐2 alloy with different undercooling. As evidenced in Figure S15 and Table S4, a larger undercooling yields a higher β phase fraction and a more uniform phase distribution, which further weakens the coupling between electrons and phonons and ultimately leads to superior thermoelectric performance. This liquid undercooling strategy demonstrates remarkable effectiveness in regulating phase composition and microstructure, offering a novel and efficient approach for the development of thermoelectric materials.

## 3 Conclusion

In summary, this study first employs rapid solidification combined with glass fluxing to achieve a large undercooling of up to Δ*T* = 122 K in the GeTe alloy. Ge enrichment effectively suppresses the formation of intrinsic *V_Ge_*, optimizes the carrier concentration to 5.7 × 10^20^ cm^−3^, and enhances the mobility to 75.3 cm^2^ V^−1^ s^−1^. Part of the high‐temperature β phase is retained to room temperature, forming an α+β dual‐phase structure with a specific crystallographic orientation relationship. This dual‐phase structure increases valley degeneracy and reduces the density‐of‐states effective mass, thereby weakening the coupling between electrical conductivity and the Seebeck coefficient and enhancing the power factor. Meanwhile, abundant phase interfaces, dislocations, twins, and point defects construct a multiscale hierarchical phonon scattering network, reducing the lattice thermal conductivity to 0.62 W·m^−1^·K^−1^ at 700 K. Consequently, the GeTe‐2 alloy achieves a peak *zT* of 1.32 at 700 K, with the entire preparation process completed in less than 2 h. This preparation method offers advantages of low cost, simple processing, and high efficiency, providing a new pathway for the preparation of high‐performance alloy‐based thermoelectric materials.

## 4 Experimental Section/Methods

The experimental process and related details are displayed in the Supporting Information.

## Author Contributions

**Guanghui Liu**: writing – original draft, investigation, validation, formal analysis, methodology, data curation. **Shuangxiong Ma**: formal analysis, methodology. **Xintong Zhang**: investigation, validation. **Dou Li**: conceptualization, writing – review and editing, methodology, resources, funding acquisition, project administration, supervision. **Weili Wang**: conceptualization, writing – review and editing, resources, funding acquisition, project administration, supervision.

## Funding

The authors are very grateful to Prof. B. Wei for his helpful discussions. This work was supported by the National Natural Science Foundation of China (No. U24A20106, 52171048, and 52501059), the China Post doctoral Science Foundation under Grant Number 2025M774344, and the Fund of the State Key Laboratory of Solidification Processing in NPU (Grant No. SKLSP202507) and the Postdoctoral Research Funding Program of Shaanxi Province (2025BSHSDZZ123).

## Conflicts of Interest

The authors declare no conflicts of interest.

## Supporting information




**Supporting File**: advs78267‐sup‐0001‐SuppMat.docx.

## Data Availability

The data that support the findings of this study are available from the corresponding author upon reasonable request.
