# Supplementary material for: Rapid Solidification Brings Ultralow Thermal Conductivity and Superior Thermoelectric Performance for Intrinsic GeTe Alloy
Source: Adv Sci (Weinh). 2026 Oct 27:e78267. Online ahead of print. doi: 10.1002/advs.78267 (PMC13713893; doi:10.1002/advs.78267)
Supplement: Supplementary file 1 [file ADVS-9999-e78267-s001.docx]

**Supporting Information**

**Rapid Solidification Brings Ultralow Thermal Conductivity and Superior Thermoelectric Performance for Intrinsic GeTe Alloy**

*Guanghui Liu, Shuangxiong Ma, Xintong Zhang, Dou Li, and Weili Wang, **

G. H. Liu, S. H. Ma, X. T. Zhang, D. Li, Prof. W. L. Wang

School of Physical Science and Technology, Northwestern Polytechnical University, Xi’an 710072, PR China

E-mail: wlwang@nwpu.edu.cn

Funding: The authors are very grateful to Prof. B. Wei for his helpful discussions. This work was supported by the National Natural Science Foundation of China (No. U24A20106, 52171048, 52501059), the China Post doctoral Science Foundation under Grant Number 2025M774344, and the Fund of the State Key Laboratory of Solidification Processing in NPU (Grant No. SKLSP202507) and the Postdoctoral Research Funding Program of Shaanxi Province (2025BSHSDZZ123).

Keywords: thermoelectricity, liquid undercooling, intrinsic GeTe, rhombohedral-cubic

**Experimental section**

*Synthesis*: High-purity raw materials: Ge and Te (99.99%, Scinor New Materials (Beijing) Technology Co., Ltd.), and B_2_O_3_ (98%, Sinopharm Group). According to the nominal compositions, the GeTe alloy was weighed as Ge_1.00_Te, Ge_1.03_Te, and Ge_1.06_Te, corresponding to GeTe-1, GeTe-2, and GeTe‑3, respectively. Here, the intrinsic GeTe alloy was prepared by rapid solidification combined with glass fluxing technologies [1-2]. First, Ge and Te raw materials together with a small amount of B_2_O_3_ glass were placed into a quartz tube, which was then inserted into a graphite sleeve. The tube was evacuated and purged with argon 2~3 times. The as-cast ingots were then loaded into new quartz tubes with glass added at both the top and bottom. The tubes were heated by a high frequency induction coil until complete melting and held for a period of time to obtain the GeTe alloy with a certain undercooling. During the above experimental process, the real-time temperature curves were monitored using a Sensor Therm-Gmbh M316 single-color pyrometer.

*Characterization*: The microstructure was analyzed using field emission scanning electron microscopy (SEM, ZEISS, Sigma 300) mounted with energy dispersive spectroscopy (EDS) operating at 20 kV. Electron Back Scatter Diffraction (EBSD, ZEISS, Sigma 300) attached to SEM was used to determine the phases composition and grain orientations. The samples were prepared by a focused ion/electron dual-beam electron microscope (FIB, Helios G4 CX), and then were determined by EM-2100F field emission transmission electron microscopy (TEM, FEI Talos F200X). Differential scanning calorimetry (DSC, STA 449 F5) was used to determine the solid-state phase transition temperatures. The crystalline phases were determined by grazing incidence X-ray diffraction (XRD, SmartLab SE) with the 2*θ* angle range of 20~70° and a scanning velocity of 3° min^-1^ using Cu Kα radiation. The molecular vibrational features were characterized using a Raman spectrometer (Raman, LabRAM HR Evolution) equipped with an ultralow frequency attachment and a 532 nm excitation laser. The chemical composition and respective chemical states of the alloy were investigated using a high-resolution X-ray photoelectron spectroscopy (XPS, ESCALAB 250Xi) system fitted with a monochromatic Al X-ray source. The room temperature Hall coefficient was characterized at 300 K using an electrical transport property measurement system (ET9105‑HS). The electrical resistivity (ρ) and Seebeck coefficient (*S*) were measured using a ZEM‑3 (Linseis LSR‑3) system with dimensions of 3^*^3^*^10 mm. κ was calculated from the relationship $\text{κ=}\text{ρD}\text{C}_{\text{P}}$, where *D* was the thermal diffusivity, $\text{C}_{\text{p}}$ was the specific heat, and $\rho$ was the mass density. The thermal diffusivity *D* was measured with a laser flash apparatus (Linseis LFA 1000) on disk‑shaped specimens of Ø12.7 mm × 2 mm thickness, while the specific heat capacity $\text{C}_{\text{p}}$ was estimated using the Dulong-Petit equation and the density *ρ* was determined by the Archimedes method.

*First-principles calculations:* First-principles calculations were performed within the framework of density functional theory as implemented in the vaspkit ab initio simulation package in this work [3-5]. The projector augmented wave method was employed to describe the interaction between ion cores and valence electrons, and the Perdew-Burke-Ernzerhof generalized gradient approximation was adopted as the exchange-correlation functional [6]. The cutoff energy was set to 480 eV, and the convergence criterion for crystal structure optimization was set to a force of less than 0.01 eV·Å^-1^ on each atom. And, a Γ-cantered Monkhorst-Pack k-point mesh was adopted for Brillouin zone sampling [7], with the mesh density adjusted according to the supercell dimensions to ensure computational accuracy and efficiency. The defect formation energies of Ge vacancies ($\text{V}_{\text{Ge}}$) in the α and β phases were calculated according to the standard supercell approach [8]. The formation energy was evaluated as a function of the chemical potentials of the constituent elements under Ge-rich and Ge-poor conditions. The differential charge density at the α/β phase interface was calculated to reveal the interfacial charge transfer and redistribution behavior. The electronic band structures and density of states were calculated along the high-symmetry paths of the Brillouin zone to analyse the band-edge dispersion and valley degeneracy of the α and β phases [9-10]. The phonon dispersion spectra were calculated using density functional perturbation theory within the harmonic approximation, and the phonon group velocities were derived from the density functional perturbation theory (DFPT) results [11-12].

**Figure S1.** Schematic diagram of the preparation process of the GeTe alloy.

**Figure S2.** EBSD micrographs of the phase map and IPF map, and pole maps of the (a) GeTe-1, (b) GeTe-2, and (c) GeTe-3 alloy.

**Table S1.** Parameters of different alloys during the preparation process.

|  | Abbreviation | Methods | | Liquidus *T* (K) | Δ*T* (K) |
| --- | --- | --- | --- | --- | --- |
| Ge_1.00_Te | GeTe-0 | Equilibrium solidification | 998 | | \ |
| Ge_1.00_Te | GeTe-1 | Rapid Solidification | 998 | | 112 |
| Ge_1.03_Te | GeTe-2 | Rapid Solidification | 1008 | | 115 |
| Ge_1.06_Te | GeTe-3 | Rapid Solidification | 1020 | | 122 |

**Figure S3.** 5 cycle DSC curves of the GeTe-1 alloy over the full heating and cooling processes.

**Figure S4.** EBSD micrographs of the (a) phase map and (b) IPF map of the GeTe-2 alloy after 5 thermal cycles.

**Figure S5.** Magnified XRD spectra of the different GeTe alloy powders in the ~30°and 41~44° range.

**Figure S6.** (a) XPS spectrums in different GeTe alloy powder. (b) Ge 2p and (c) Te 3d core level spectrum of the GeTe-2 alloy.

**Figure S7.** SEM image and EDS spectrums of the (a) GeTe-1, (b) GeTe-2, and (c) GeTe-3 alloy.

**Figure S8.** The STEM sample is prepared by focused Ion beam (FIB) ultrathin sectioning.

**Figure S9.** (a) HAADF-STEM images, (b, c) elemental mapping of Ge and Te and (d) Corresponding EDS map in the GeTe-2 alloy.

**Figure S10.** Density of states (DOS) of the (a) α phase and (b) β phase.

**Figure S11.** Pisarenko plot showing the Seebeck coefficient (*S*) as a function of carrier concentration (*n*).

**Figure S12.** Differential charge density calculations of the α/β phase interface.

**Figure S13.** The electronic thermal conductivity ($\text{κ}_{\text{e}}$) of the GeTe alloy.

**Figure S14.** $\text{zT}_{\text{ave}}$ within 300~750 K of the GeTe alloy.

**SPB model for the Lorenz number**

Within the SPB model with acoustic phonon scattering, the Lorenz number (*L*) is given by [8]:

$$\text{L}\text{=}{\text{(}\frac{\text{κ}_{\text{B}}}{\text{e}}\text{)}}^{\text{2}}\left\{ \frac{\text{(r+7/2)}\text{F}_{\text{r+5/2}}\text{(}\text{η}\text{)}}{\text{(r+3/2)}\text{F}_{\text{r+1/2}}\text{(}\text{η}\text{)}}\text{-}\left[ \frac{\text{(r+5/2)}\text{F}_{\text{r+3/2}}\text{(}\text{η}\text{)}}{\text{(r+3/2)}\text{F}_{\text{r+1/2}}\text{(}\text{η}\text{)}} \right]^{\text{2}} \right\}$$

where $\text{κ}_{\text{B}}$ is the Boltzmann constant, *e* is the electron charge, *r* is the scattering factor, and $\text{η}\text{=}\text{E}_{\text{F}}\text{/}\text{κ}_{\text{B}}\text{T}$ is the reduced Fermi energy. The Fermi integral is:

$$\text{F}_{\text{n}}\text{(}\text{η}\text{)=}\int_{\text{0}}^{\text{∞}} \frac{\text{x}^{\text{n}}}{\text{1+exp}\text{(}\text{x}\text{-η}\text{)}}\text{d}\text{x}$$

The reduced Fermi energy $\eta$ is obtained from the experimentally measured Seebeck coefficient (*S*) through:

$$\text{S}\text{=}\frac{\text{κ}}{\text{e}}\text{(}\frac{\text{2}\text{F}_{\text{1}}}{\text{F}_{\text{0}}}\text{-}\text{η}\text{)}$$

Here $\text{r}\text{=-1/2}$ for acoustic phonon scattering. The temperature-dependent *L* is therefore calculated from the measured *S(T)* at each temperature point rather than assuming a constant metallic limit. Estimate $\text{m}^{\text{*}}$ using $\text{η}$:

$$\text{n}\text{=4π}{\text{(}\frac{\text{2}\text{m}^{\text{*}}\text{kT}}{\text{h}^{\text{2}}}\text{)}}^{\text{3/2}}\text{F}_{\text{1/2}}$$

The Hall carrier concentration ($\text{n}_{\text{H}}\text{=1/}\text{R}_{\text{H}}\text{e}$) is related to the chemical carrier concentration *n* *via* $\text{n}_{\text{H}}\text{=}\text{n}\text{/}\text{r}_{\text{H}}$, where the Hall factor ($\text{r}_{\text{H}})$ for acoustic phonon scattering is given by:

$$\text{r}_{\text{H}}\text{=}\frac{\text{3}}{\text{2}}\text{F}_{\text{1/2}}\frac{\text{F}_{\text{-1/2}}}{\text{2}\text{F}_{\text{0}}^{\text{2}}}$$

Calculate the Lorenz number (*L*), using the following SPB equation or a plot of *L* versus $\text{S}$.

$$\text{L}\text{=}\frac{\text{k}^{\text{2}}}{\text{e}^{\text{2}}}\frac{\text{3}\text{F}_{\text{0}}\text{F}_{\text{2}}\text{-4}\text{F}_{\text{1}}^{\text{2}}}{\text{F}_{\text{0}}^{\text{2}}}$$

Estimate the electronic contribution $\text{κ}_{\text{e}}$ to the thermal conductivity. Subtract this from the total thermal conductivity and obtain an estimate for the lattice contribution $\text{κ}_{\text{l}}$:

$$\text{κ}_{\text{l}}\text{=}\text{κ}\text{-}\text{κ}_{\text{e}}\text{=}\text{κ}\text{-}\text{L}\text{σ}\text{T}$$

In this work, the SPB model is used to calculate the temperature-dependent *L* from the measured *S(T)* at each temperature point, and the results are compared with the empirical formula to ensure reliability [8, 13-14]. The calculated *L* decreases from about 2.38×10^-8^ W·Ω·K^-2^ at 300 K to approximately 1.64×10^-8^ W·Ω·K^-2^ at 300 K, significantly lower than the degenerate limit of 2.4×10^-8^ W·Ω·K^-2^ (Table S3). The formulas and parameters used in the calculations are provided in the Supporting Information.

**Table S2.** Calculation of temperature-dependent Lorenz Number (*L*) from measured *S(T)* at each temperature point.

| GeTe-0 | | GeTe-1 | | GeTe-2 | | GeTe-3 | |
| --- | --- | --- | --- | --- | --- | --- | --- |
| S  (μ·V·K^-1^) | *L* (10^-8^ W·Ω·K^-2)^ | S  (μ·V·K^-1^) | *L* (10^-8^ W·Ω·K^-2)^ | S  (μ·V·K^-1^) | *L* (10^-8^ W·Ω·K^-2)^ | S  (μ·V·K^-1^) | *L* (10^-8^ W·Ω·K^-2)^ |
| 25.91 | 2.38 | 31.81 | 2.34 | 30.51 | 2.35 | 31.45 | 2.35 |
| 32.06 | 2.34 | 35.91 | 2.32 | 41.79 | 2.28 | 42.04 | 2.28 |
| 40.18 | 2.29 | 45.04 | 2.26 | 52.55 | 2.20 | 53.64 | 2.19 |
| 51.08 | 2.21 | 58.35 | 2.16 | 65.43 | 2.11 | 65.88 | 2.11 |
| 64.55 | 2.12 | 75.63 | 2.05 | 83.32 | 2.00 | 86.13 | 1.99 |
| 82.37 | 2.01 | 96.56 | 1.93 | 105.95 | 1.88 | 111.60 | 1.86 |
| 103.85 | 1.89 | 120.36 | 1.82 | 132.01 | 1.78 | 143.90 | 1.74 |
| 127.66 | 1.80 | 140.53 | 1.75 | 158.14 | 1.70 | 183.19 | 1.65 |
| 139.80 | 1.76 | 154.38 | 1.71 | 175.21 | 1.66 | 196.52 | 1.63 |
| 147.59 | 1.73 | 170.23 | 1.68 | 189.89 | 1.64 | 196.28 | 1.63 |

**Phonon modeling studies**

The spectral lattice thermal conductivity (*κ_s_*) is calculated by the Debye-Callaway model [15-16], the *κ_s_* can be expressed by:

$$\text{κ}_{\text{s}}\text{=}\frac{\text{k}_{\text{B}}}{\text{2}\text{π}^{\text{2}}\text{v}_{\text{s}}}{\text{(}\frac{\text{k}_{\text{B}}\text{T}}{\text{ћ}}\text{)}}^{\text{3}}\text{τ}_{\text{tot}}\frac{\text{z}^{\text{4}}\text{e}^{\text{z}}}{{\text{(}\text{e}^{\text{z}}\text{-1)}}^{\text{2}}}$$

Where $\text{k}_{\text{B}}$ is the Boltzmann constant, $\text{ν}_{\text{s}}$ is the average sound velocity, *ћ* is the reduced Planck constant, $\text{z}\text{=}\text{ω}\text{=}\text{k}_{\text{B}}\text{T}$ (*ω* denoting the phonon frequency) is the reduced phonon frequency and $\text{τ}_{\text{tot}}$ is the total relaxation time, namely:

$$\text{τ}_{\text{tot}}^{\text{-1}}\text{=}\text{τ}_{\text{B}}^{\text{-1}}\text{+}\text{τ}_{\text{U}}^{\text{-1}}\text{+}\text{τ}_{\text{DS}}^{\text{-1}}\text{+}\text{τ}_{\text{DC}}^{\text{-1}}\text{+}\text{τ}_{\text{PD}}^{\text{-}\text{1}}$$

The total relaxation time is contributed from each scattering process of grain boundaries scattering (B), Umklapp scattering (U), dislocation strain field scattering (DS), dislocation core scattering (DC) and point defects scattering (PD), corresponding relaxation time are:

$$\text{τ}_{\text{B}}^{\text{-1}}\text{=}\frac{\text{v}_{\text{s}}}{\text{G}}$$

$$\text{τ}_{\text{U}}^{\text{-1}}\text{=}\frac{\text{ћ}\text{γ}^{\text{2}}}{\bar{\text{M}}{\text{v}_{\text{s}}}^{\text{2}}\text{θ}_{\text{D}}}\text{ω}^{\text{2}}\text{T}\text{ }\text{exp(-}\frac{\text{θ}_{\text{D}}}{\text{3}\text{T}}\text{)}$$

$$\text{τ}_{\text{DS}}^{\text{-1}}\text{=0.6}\text{B}_{\text{D}}^{\text{2}}\text{N}_{\text{D}}\text{γ}^{\text{2}}\text{ω}\left[ \frac{\text{1}}{\text{2}}\text{+}\frac{\text{1}}{\text{24}}\text{(}\frac{\text{1-2}\text{v}}{\text{1-}\text{v}}\text{)}{\text{(1+}\sqrt{\text{2}}\text{(}\frac{\text{v}_{\text{l}}}{\text{v}_{\text{t}}}\text{))}}^{\text{2}} \right]$$

$$\text{τ}_{\text{DC}}^{\text{-1}}\text{=}\text{N}_{\text{D}}\frac{\text{V}_{\text{a}}^{\text{4/3}}}{\text{V}_{\text{a}}^{\text{2}}}\text{ω}^{\text{3}}$$

$$\text{τ}_{\text{PD}}^{\text{-1}}\text{=}\frac{\bar{\text{V}}\text{ω}^{\text{4}}}{\text{4π}{\text{v}_{\text{s}}}^{\text{3}}}\text{Γ}$$

**Table S3.** Parameters for phonon modeling studies (the GeTe-1 alloy).

| Parameters | Values |
| --- | --- |
| Debye temperature $\Theta_{D}$ (K) | 196 |
| Ratio between normal and Umklapp process *β* | 2.5 (fitted) ^[15]^ |
| Longitudinal sound velocity $v_{L}$ (m·s^-1^) | 3165 |
| Transverse sound velocity $v_{T}$ (m·s^-1^) | 1775 |
| Sound velocity *v* （m·s-1） | 1972 |
| Average atomic mass $\bar{M}$ (kg) | 1.6 6×10^-25^ |
| Average atomic volume $\bar{V}$ (m^3^) | 2.68×10^-29^ |
| Grain size *d* (μm) | 5 |
| Point defect scattering parameter *Γ* | 0.18 (fitted) ^[15]^ |
| Grüneisen parameter *γ* | 1.5 |
| Lattice parameter *a* (Å) | 6.06 |
| Density of dislocation (cm^-2^) | 5.6×10^11^ |
| Magnitude of Burger’s vector $B_{D}$ (Å) | 4.23 |
| Number of stacking faults crossing a line of unit length $N_{S}$ (cm^-1^) | 4×10^5^ |
| Average domain width (nm) | 200 |
| Domain fitting parameter | 0.1 (fitted) ^[15]^ |

**Figure S15.** EBSD micrographs of the (a) phase map and (b) IPF map of the GeTe alloy with Δ*T*=35 K.

**Table S4.** Parameters of different alloys during the preparation process.

| Δ*T*  (K) | *T*  (K) | *S*  (μV·K^-1^) | *σ*  (×10^4^ S·cm^-1^) | *PF*  (μW·cm^-1^·K^-2^) | *L*  (×10^-8^ W·Ω·K^-2^) | $\text{κ}_{\text{tot}}$  (W·m^-1^·K^-1^) | $\text{κ}_{\text{e}}$  (W·m^-1^·K^-1^) | $\text{κ}_{l}$  (W·m^-1^·K^-1^) | *zT* |
| --- | --- | --- | --- | --- | --- | --- | --- | --- | --- |
| 35 | 700 | 175.21 | 1.67 | 51.47 | 1.66 | 2.71 | 1.99 | 0.72 | 1.32 |
| 115 | 700 | 152.70 | 1.69 | 39.45 | 1.71 | 3.10 | 2.08 | 1.02 | 0.90 |

**References**

1. X. L. Zhao, J. Chang, M. J. Lin, et al., Liquid State Property and Rapid Peritectic Solidification of Refractory Mo-33.3 At.% Zr Alloy under Electrostatic Levitation Condition, *Acta Materialia* 281 (2024), https://doi.org/10.1016/j.actamat.2024.120401.

2. X. J. Han, C. Yang, B. Wei, et al., Rapid Solidification of Highly Undercooled Ni-Cu Alloys, *Materials Science and Engineering A* 307 (2001): 35, https://doi.org/10.1016/s0921-5093(00)01969-9.

3. G. Kresse, Efficient Iterative Schemes for ab Initio Total-Energy Calculations Using a Plane-Wave Basis Set, *Physical Review B* 54 (1996): 11169, https://doi.org/10.1103/PhysRevB.54.11169.

4. G. Kresse, J. Furthmiiller, Efficiency of ab-Initio Total Energy Calculations for Metals and Semiconductors Using a Plane-Wave Basis Set, *Computational Materials Science* 6 (1996): 15, https://doi.org/10.1016/0927-0256(96)00008-0.

5. V. Wang, N. Xu, J.-C. Liu, et al., Vaspkit: A User-Friendly Interface Facilitating High-Throughput Computing and Analysis Using Vasp Code, *Computer Physics Communications* 267 (2021): 108033, https://doi.org/10.1016/j.cpc.2021.108033.

6. J. Heyd, G. E. Scuseria, Efficient Hybrid Density Functional Calculations in Solids: Assessment of the Heyd-Scuseria-Ernzerhof Screened Coulomb Hybrid Functional, *The Journal of Chemical Physics* 121 (2004): 1187, https://doi.org/10.1063/1.1760074.

7. H. J. Monkhorst, J. D. Pack, Special Points for Brillouin-Zone Integrations, *Physical Review B* 13 (1976): 5188, https://doi.org/10.1103/PhysRevB.13.5188.

8. M. Zhang, Z. Gao, Q. Lou, et al., Achieving High Carrier Mobility and Thermal Stability in Plainified Rhombohedral GeTe Thermoelectric Materials with zT > 2, *Advanced Functional Materials* 34 (2023): 2307864, https://doi.org/10.1002/adfm.202307864.

9. X. Liu, W. Wang, Y. Wang, et al., Realizing High Thermoelectric Performance in GeTe‐Based Supersaturated Solid Solutions, *Advanced Energy Materials* 14 (2024): 2304029, https://doi.org/10.1002/aenm.202304029.

10. J. Zhu, R. Li, X. Tan, et al., Valence Band Modification and Enhanced Phonon‐Phonon Interactions for High Thermoelectric Performance in GeTe, *Advanced Functional Materials* 35 (2024): 2417260, https://doi.org/10.1002/adfm.202417260.

11. S. Baroni, S. De Gironcoli, A. Dal Corso, Phonons and Related Crystal Properties from Density-Functional Perturbation Theory, *Reviews of Modern Physics* 73 (2001): 515, https://doi.org/10.1103/revmodphys.73.515.

12. K. Xu, Y. Fu, W. Zuo, et al., Phase-Stabilized GeTe with Optimized Interfaces for High-Performance Thermoelectric Energy Conversion, *Energy & Environmental Science* 18 (2025): 9274, https://doi.org/10.1039/d5ee04065e.

13. T. Xing, C. Zhu, Q. Song, et al., Ultralow Lattice Thermal Conductivity and Superhigh Thermoelectric Figure-of-Merit in (Mg, Bi) Co-Doped GeTe, *Advanced Materials* 33 (2021): 2008773, https://doi.org/10.1002/adma.202008773.

14. J. Yu, X. Liu, Y. Jiang, et al., Giant Zt Enhancement in Rhombohedral GeTe-Based Thermoelectric Materials, *Nature Communications* 17 (2026): 4000, https://doi.org/10.1038/s41467-026-70793-6.

15. Y. Jiang, J. Dong, H.-L. Zhuang, et al., Evolution Ofdefect Structures Leading Tohigh zT in GeTe-Based Thermoelectric Materials, *Nature Communications* 13 (2022): 6087 https://doi.org/10.1038/s41467-022-33774-z.

16. Y. Jin, Y. Qiu, S. Bai, et al., Modifying Roles of CuSbSe_2_ in Realizing High Thermoelectric Performance of GeTe, *Advanced Energy Materials* 14 (2024): 2400623, https://doi.org/10.1002/aenm.202400623.
